# Combining Laser-Induced Breakdown Spectroscopy and Visible Near-Infrared Spectroscopy for Predicting Soil Organic Carbon and Texture: A Danish National-Scale Study

**DOI:** 10.3390/s24144464

**Published:** 2024-07-10

**Authors:** Alex Wangeci, Daniel Adén, Thomas Nikolajsen, Mogens H. Greve, Maria Knadel

**Affiliations:** 1 Department of Agroecology, Aarhus University, Blichers Allé 20, 8830 Tjele, Denmark; 2 FOSS Analytical A/S, Nils Foss Allé 1, 3400 Hillerød, Denmark; 3 GeoPark Vestjylland, Skæreum Møllevej 4, 7570 Vemb, Denmark

**Keywords:** soil organic carbon, texture, PLSR, iPLSR, data fusion, variable selection

## Abstract

Laser-induced breakdown spectroscopy (LIBS) and visible near-infrared spectroscopy (vis-NIRS) are spectroscopic techniques that offer promising alternatives to traditional laboratory methods for the rapid and cost-effective determination of soil properties on a large scale. Despite their individual limitations, combining LIBS and vis-NIRS has been shown to enhance the prediction accuracy for the determination of soil properties compared to single-sensor approaches. In this study, we used a comprehensive Danish national-scale soil dataset encompassing mostly sandy soils collected from various land uses and soil depths to evaluate the performance of LIBS and vis-NIRS, as well as their combined spectra, in predicting soil organic carbon (SOC) and texture. Firstly, partial least squares regression (PLSR) models were developed to correlate both LIBS and vis-NIRS spectra with the reference data. Subsequently, we merged LIBS and vis-NIRS data and developed PLSR models for the combined spectra. Finally, interval partial least squares regression (iPLSR) models were applied to assess the impact of variable selection on prediction accuracy for both LIBS and vis-NIRS. Despite being fundamentally different techniques, LIBS and vis-NIRS displayed comparable prediction performance for the investigated soil properties. LIBS achieved a root mean square error of prediction (RMSEP) of <7% for texture and 0.5% for SOC, while vis-NIRS achieved an RMSEP of <8% for texture and 0.5% for SOC. Combining LIBS and vis-NIRS spectra improved the prediction accuracy by 16% for clay, 6% for silt and sand, and 2% for SOC compared to single-sensor LIBS predictions. On the other hand, vis-NIRS single-sensor predictions were improved by 10% for clay, 17% for silt, 16% for sand, and 4% for SOC. Furthermore, applying iPLSR for variable selection improved prediction accuracy for both LIBS and vis-NIRS. Compared to LIBS PLSR predictions, iPLSR achieved reductions of 27% and 17% in RMSEP for clay and sand prediction, respectively, and an 8% reduction for silt and SOC prediction. Similarly, vis-NIRS iPLSR models demonstrated reductions of 6% and 4% in RMSEP for clay and SOC, respectively, and a 3% reduction for silt and sand. Interestingly, LIBS iPLSR models outperformed combined LIBS-vis-NIRS models in terms of prediction accuracy. Although combining LIBS and vis-NIRS improved the prediction accuracy of texture and SOC, LIBS coupled with variable selection had a greater benefit in terms of prediction accuracy. Future studies should investigate the influence of reference method uncertainty on prediction accuracy.

## 1. Introduction

Soil quality assessments are an important step in determining the ability of a soil to support crop development. Soil quality indicators, which include physical, chemical, and biological attributes, interact in a complex way, influencing the overall soil function. Soil organic carbon (SOC) cuts across the physical, chemical, and biological attributes, while soil texture (i.e., the percentage of clay, silt, and sand content) is a physical parameter [1]. The continuous monitoring of these soil quality indicators has become a necessity to guarantee optimal productivity and sustainability. Indeed, notable efforts to promote soil health have been made by the European Union. For instance, a soil monitoring law was recently proposed to harmonize the definition of soil health, therefore enabling sustainable soil management and remediation of contaminated sites [2]. In this law, SOC and texture, among other soil properties, have been listed as estimators of soil descriptor values.

The steady development of spectroscopic techniques as alternatives to wet chemistry methods has led to improved, cheaper, and faster analyses in the laboratory and in the field. More importantly, their application in agriculture, such as in the determination of soil properties, has enabled faster decision making, and thus, timely agronomic interventions. Laser-induced breakdown spectroscopy (LIBS) and visible near-infrared spectroscopy (vis-NIRS) stand out as potential candidates poised to revolutionize soil analysis. Despite the fact that the vis-NIRS technique is already an established method for soil analysis, LIBS for soil applications is still in its infancy. Nonetheless, there are several studies that used both techniques for the determination of different soil properties [3,4,5,6].

LIBS is an elemental technique suitable for determining elemental composition in a sample. For instance, in soil analysis, phosphorus, cadmium, or lead can be determined through a direct relationship with the emission lines related to the three elements. On the other hand, for the determination of properties like SOC and texture, an indirect association with multiple emission lines related to the investigated properties, such as the C emission lines, as well as emission lines linked to clay and sand minerals [3,7], can be used for the quantitative analysis of SOC. The texture content can be associated with the relative abundance of Si, Al, Mg, and Fe emission lines to sand, silt, and clay mineralogy [8]. The ability of LIBS to distinguish distinct elemental emission lines could potentially enable the generalization of prediction models covering vast geographical distributions. However, the elemental complexity of soil presents the challenge of resolving the emission lines of elements occupying the same wavelength range. This challenge necessitates the use of higher-resolution spectrometers, leading to a compromise between spectral resolution and wavelength range. As a result, LIBS has become a more complex and expensive system compared to vis-NIRS [9].

On the other hand, vis-NIRS is a molecular technique suitable for determining spectrally active compounds in a sample. For soil analysis, spectrally active compounds include clay mineralogy, water, and organic matter (OM) [10]. The difference in the size of soil particles can influence light scattering, thus complementing the spectrally active clay mineralogy to accurately determine soil texture [11,12]. SOC is related to SOM, thus the absorbance peaks in the visible regions associated with OM [13] can be used for the determination of SOC. Vis-NIRS is characterized by weak peaks originating from overtones and combinations of fundamental vibrations, a challenge compounded by absorption features that are not unique to specific soil properties which often overlap [14,15]. This could, in turn, negatively impact the prediction of soil properties, especially for different types of soils from different geological origins.

Various studies have used LIBS and vis-NIRS, separately and in combination, to determine SOC and texture. Notably, SOC has received special attention, as demonstrated by the numerous studies focusing on predicting SOC using LIBS and/or vis-NIRS [14,16,17]. On the other hand, soil texture has not received as much attention, especially for the newer LIBS technique. Although texture is a relatively stable soil property that is not bound to drastically change over time, improving the predictions using proximal sensors is necessary, especially because the alternative wet chemistry methods (e.g., pipette and hydrometer methods) are extremely long and tedious. A promising strategy to improve the prediction accuracy of soil properties is the combination of two or more spectroscopic techniques. Already, there have been improved predictions for various soil properties reported when LIBS and vis-NIRS measurements were combined [9,14,18]. For instance, Bricklemyer, Brown, Turk and Clegg [14] reported improved total carbon predictions for combined LIBS-vis-NIRS predictions as compared to predictions from single sensors. However, the authors noted that SOC and inorganic C did not benefit from the data fusion technique. Instead, there was a higher vis-NIRS prediction accuracy for SOC and higher prediction accuracy for LIBS inorganic C. The challenge of the availability of a large dataset covering greater variability in terms of the range of the investigated soil properties has been previously reported by several studies where LIBS and vis-NIRS were combined for predicting soil properties [9,14,18]. In all of the above-mentioned studies, there was a convergent agreement on the need to investigate the benefit of data fusion on a large-scale dataset covering a wide range of soil properties.

Nonetheless, it is also important to evaluate the benefit of combining the two spectroscopic techniques by comparing the relative improvement in contrast to the single-sensor technique. This can also be assessed by exploring improved data analysis techniques, such as variable selection, to reduce the number of input variables used for developing the prediction models. Several studies have demonstrated the added benefit of using variable selection for predicting SOC and/or texture for LIBS [14,19] and vis-NIRS [20,21,22]. However, it has also been reported that variable selection does not always improve prediction accuracy when compared to prediction models developed using the full spectrum. For instance, when applying LIBS for predicting texture, an overall higher accuracy of PLSR was reported as compared to variable selection using an elastic net [19]. In another study, similar or only slight improvements were obtained when feature selection algorithms were used for estimating SOC using vis-NIRS [23].

In this study, samples covering a national scale were used to assess the effect of combining LIBS and vis-NIRS for predicting SOC and texture. The dataset accounts for different soil types, textural classes, high concentration range of SOC and other properties, different land use practices, and soil profiles. Prediction models from a combination of both techniques were developed and compared, based on prediction accuracy, with the individual sensor techniques. Finally, PLSR (with variable selection) models were developed and compared with the individual and combined LIBS-vis-NIRS models.

## 2. Materials and Methods

### 2.1. Soil Samples and Analysis

A total of 1110 samples collected from Denmark were used for this study. The dataset comprised topsoil (0–20 cm) and subsoil (20–30, and all the way to 200 cm at 10 cm thickness intervals) samples representing both agricultural and nonagricultural land uses. The land use was defined based on the vegetation cover recorded during sampling. Approximately 30% of the dataset comprised topsoils, while 63% were soils collected from agricultural sites. The soil types represented include Alisols, Anthrosols, Arenosols, Cambisols, Fluvisols, Gleysols, Histosols, Luvisols, Phaeozems, Podzols, and Regosols [24,25,26].

The samples were air-dried and sieved to <2 mm and stored under cool and dark conditions to mitigate against any physical or chemical changes that could occur over time.

The soil texture (clay, silt, and sand content) was determined via a combination of wet sieving and hydrometer methods [27], while the SOC content was determined via dry combustion using a LECO CN-2000 instrument (LECO Corp., St. Joseph, MI, USA) after the subtraction of carbonates, where present.

**Figure 1:**
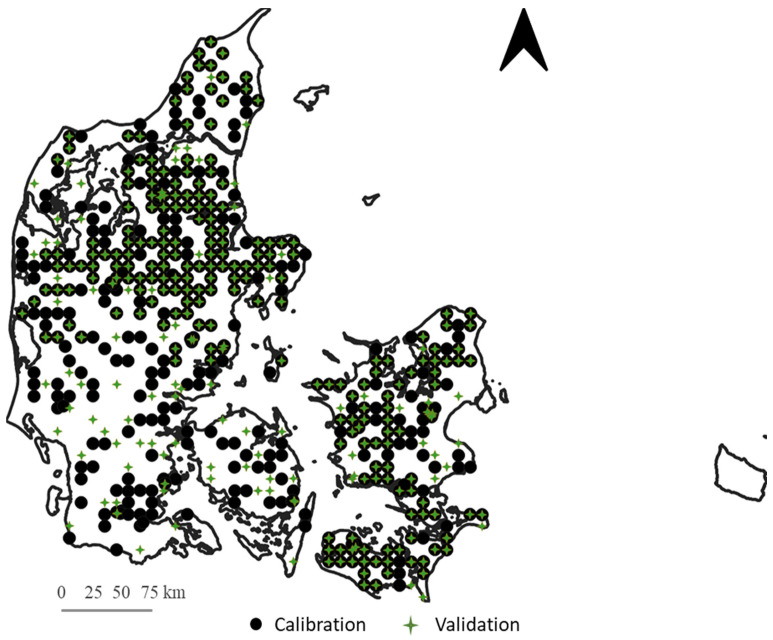
Distribution across Denmark of the 1110 samples used in this study. Calibration and validation samples are indicated.

The dataset was manually split into calibration and validation samples. Here, every third sample was included in the validation set, and the remaining samples were designated the calibration set. This resulted in a total of 739 calibration and 371 validation samples that covered the entire of Denmark (Figure 1).

### 2.2. LIBS Measurement

The LIBS system used was a commercial LIBS instrument, Micral^TM^ (FOSS, Hillerød, Denmark). A detailed description of the setup is available in Wangeci, Adén, Greve and Knadel [7]. Briefly, the system consists of a microchip neodymium-doped yttrium aluminum garnet (Nd: YAG) laser operating at a wavelength of 1064 nm with a pulse energy of 0.15 mJ, a pulse duration of 1.5 ns, and a repetition rate of 200 Hz. The laser beam was focused through a lens to minimize spot size and increase the irradiance (which was approximately 1.3 × 10^11^ W/cm^2^). The focusing lens was an anti-reflective (AR)-coated achromatic doublet and a 40 ± 0.4 mm focal length, which yielded a root mean square (RMS) spot diameter of approximately 10 µm. The integration time was 6.7 ms, starting simultaneously with the laser pulse. Thus, at a 200 Hz repetition rate, each sub-spectrum covered two full plasma lifetimes.

The LIBS spectrum was acquired in the wavelength range between 174 and 427 nm at a spectral resolution of 0.1 nm, resulting in 2491 variables. For soil measurements, approximately 1 g of the soil sample was placed into a 14 mm diameter sample cup and pelletized using an automatic press (FOSS, Hillerød, Denmark) at 1948 kg cm^−2^ for 30 s. This resulted in a flat-surface sample pellet that was then presented for LIBS measurement. The sample pellet was rotated in a spiral movement during measurement, providing a fresh sample surface for each laser pulse. A total of 3000 spectra were averaged for each sample measurement. This mitigated the effect of pellet surface heterogeneity that could influence the LIBS signal due to shot-to-shot variability [28]. To enable the study of LIBS signal for wavelengths shorter than approximately 190 nm, the full optical path was continuously purged using nitrogen gas during the measurements.

### 2.3. Vis-NIRS Measurement

The vis-NIRS system used was a commercial vis-NIRS instrument, NIRS^TM^ DS2500 (FOSS, Hillerød, Denmark). The instrument covered a spectral range from 400 to 2500 nm and a spectral resolution of 0.5 nm. Approximately 50 g of the soil sample was placed into a 7 cm sample cup that has a 6 cm quartz window mounted at the bottom (approximately 28 cm^2^ sampling area). The average of the spectra collected from seven different spots was used for subsequent spectral analysis. In this case, the absorbance, defined as A = [log(1/R)], where R is the reflectance, was used.

### 2.4. Data Analysis

#### 2.4.1. Partial Least Square Regression

All multivariate data analyses were carried out in MatLab R2021a (MathWorks, Inc., Natick, MA, USA) and PLS Toolbox 8.7 software (Eigenvector Research Inc., Manson, WA, USA).

We used partial least square regression (PLSR) to develop prediction models for each of the investigated soil properties. PLSR is a multivariate data analysis method that aims to predict Y from X and to describe their common structure, where Y is an n observation by m variables response, and X is an n observation by p variables predictor [29].

Before PLSR, the Automatic Whittaker Filter baseline correction method [30] and mean-centering were selected for LIBS spectral preprocessing, while standard normal variate (SNV) and mean-centering were selected for vis-NIRS.

For each model, the optimal number of latent variables (LVs) was selected to minimize the risk of overfitting. The selection of optimal LVs was determined by inspecting the plot of root mean square error (RMSE) versus the number of latent variables, then selecting the number of latent variables where the minimum RMSE was achieved without increasing the distance between the RMSE of calibration (RMSEC) and the RMSE of cross-validation (RMSECV) [31].

We used the ratio of performance to interquartile distance (RPIQ) to compare the performance of the prediction models for each of the investigated soil properties. The RPIQ is a dimensionless measure that takes the interquartile range into account, thus enabling a comparison of performance across soil properties. The higher the RPIQ, the better the prediction model.

(1) $$\mathrm{RMSE}=\sqrt{\frac{\sum_{i=1}^{N}{\left(y_{i}-{\hat{y}}_{i}\right)}^{2}}{N}}$$

where *y_i_* and ${\hat{y}}_{i}$ is the *i*th measured value and corresponding predicted value of the soil property, respectively, and *N* is the total number of samples.

(2) $$\mathrm{RPIQ}=\frac{\left(Q3-Q1\right)}{RMSEP}$$

where *Q*1 and *Q*3 are the first and third quartiles, respectively. The difference between the two represents the interquartile range.

#### 2.4.2. Variable Selection

To reduce the number of variables and simplify the model, a variable selection algorithm method that applies interval partial least square regression (iPLSR) was used to select important regions in the soil spectra. Briefly, the iPLSR algorithm divides the entire emission spectral data into a few intervals and then applies the PLSR model separately to each model [32]. Ideally, iPLSR identifies important intervals that result in better models when applied in the PLSR (as compared to using the full wavelength range). Since the interval size can influence the accuracy of the calibration model [20], we tested different interval sizes (8, 10, 20, 30, 40, and 50). We then used the full cross-validation model results to select the interval size that produced the best model (lowest RMSECV).

#### 2.4.3. Data Fusion

LIBS and vis-NIRS are two different techniques whose signal outputs differ in terms of scale. LIBS spectral output (photons) is expressed as counts at the respective wavelengths. On the other hand, vis-NIRS measures reflectance, which is often converted to absorbance. The absorbance scale ranges from 0 to 1. Therefore, to combine the two techniques, the spectra from LIBS and vis-NIRS were independently preprocessed. Automatic Whittaker Filter baseline correction method was applied to the LIBS spectra, while SNV was applied to the vis-NIRS spectra. Next, the spectra from each technique were decomposed using principal component analysis (PCA), and the PC scores were mean-centered and variance-scaled before merging into a single combined LIBS-vis–NIRS predictor dataset [33]. For both LIBS and vis-NIRS, 20 PCs were used, for a total of 40 input variables.

#### 2.4.4. Correlation between LIBS and vis-NIRS Models

The PLSR models for LIBS and vis-NIRS prediction were used to evaluate the correlation between the two techniques. Using the validation results, the predicted values for LIBS and vis-NIRS were plotted against each other for clay, silt, sand, and SOC. The calculated coefficient of determination (R^2^) was used to compare and determine the extent of the relationship between LIBS and vis-NIRS.

#### 2.4.5. Regression Vector Analysis

To understand the emission lines (for LIBS) and absorption bands (for vis-NIRS) explaining most of the variability of the soil properties, we performed a regression vector analysis. The regression vector analysis involves examining the scores assigned to the original input variables, thus deducing their influence on the model.

## 3. Results and Discussion

### 3.1. Exploratory Data Analysis

**Figure 2:**
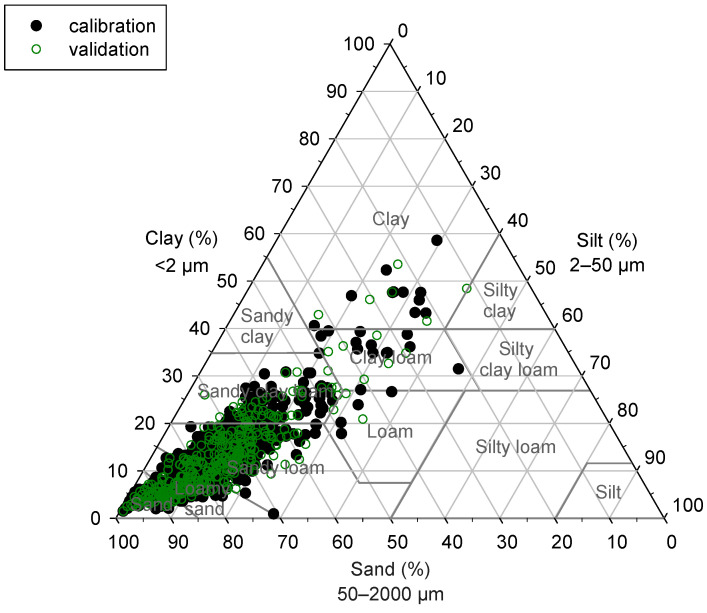
Texture distribution of the 1110 samples used in the study (USDA). The calibration and validation samples are indicated.

There was a high variability in terms of the content of the investigated soil properties. The clay content ranged from 1 to 59%, silt 0 to 46%, sand 12 to 98%, and SOC 0.01 to 5.92%. Most Danish soils are generally sandy [24], and this was also visible in our dataset (Figure 2). As the variability of soil properties influences model performance [13,34], we used the coefficient of variation (CV) to verify that the same variability was represented in both the calibration and validation set. The overall CV ranged from 128% to 133% (Table 1).

**Figure 3:**
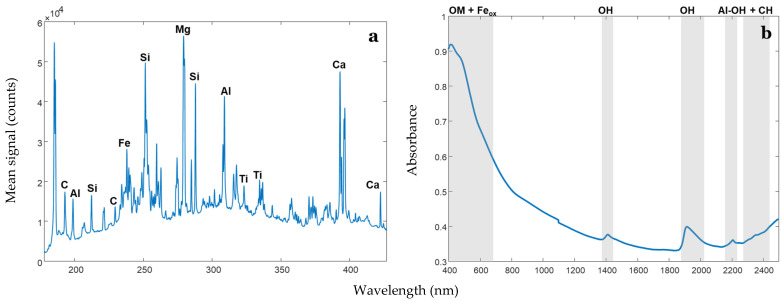
A typical mean raw LIBS soil spectrum (**a**) as observed in the 174 to 430 nm wavelength range, and a vis-NIRS soil spectrum (**b**) as observed in the visible and near-infrared range. Common emission lines (for LIBS) and absorption bands (for vis-NIRS) are indicated.

Figure 3 shows a typical LIBS spectrum and a typical vis-NIRS soil spectrum. Common emission lines (LIBS) and absorption bands (vis-NIRS) are indicated.

### 3.2. Prediction Models

PLSR models were developed for LIBS, vis-NIRS, and combined LIBS-vis-NIRS to correlate the spectra and the measured soil property content. We used the RPIQ to compare the performance of the prediction models across the investigated soil properties for the single-sensor and combined predictions (Table 2). The RMSECVs and RMSEPs for texture were rounded off to the nearest integer since data from wet chemistry are usually presented without the fractional part.

#### 3.2.1. Single-Sensor Predictions

For LIBS, the clay prediction model showed the highest accuracy (RPIQ = 2.6), followed by sand and SOC, which were comparable (RPIQ = 2.5), and silt (RPIQ = 2.3). Likewise, for the vis-NIRS prediction models, clay had the highest accuracy (RPIQ = 2.8), followed by SOC (RPIQ = 2.4), sand (RPIQ = 2.3), and silt prediction models (RPIQ = 2.0).

**Figure 4:**
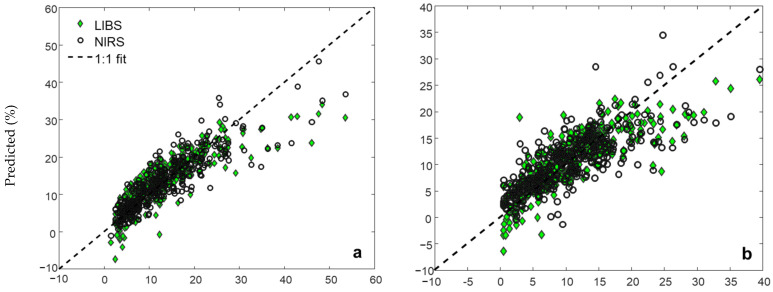

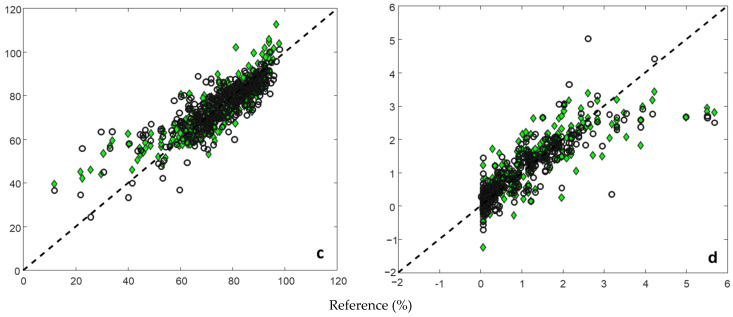
Regression plots for LIBS and vis-NIRS validation models for clay (**a**), silt (**b**), sand (**c**), and SOC (**d**).

The regression model plots for LIBS and vis-NIRS were largely similar (Figure 4). There was a slightly higher number of samples underpredicted for the LIBS model, notably for the low and high-silt-content samples (Figure 4b). There was also a notable underprediction for higher clay (>30%) and SOC (>3.5%) samples by both LIBS and vis-NIRS prediction models. On the other hand, lower-sand-content samples (<50%) were overpredicted by both techniques (Figure 4c).

#### 3.2.2. Combined LIBS-vis-NIRS Models

**Figure 5:**
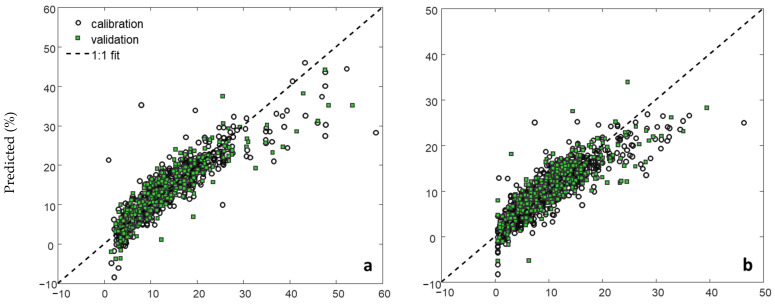

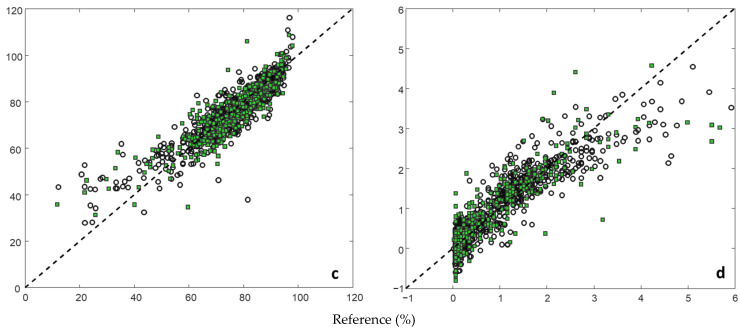
Regression plots for combined LIBS-vis-NIRS models for clay (**a**), silt (**b**), sand (**c**), and SOC (**d**).

There was a higher prediction accuracy for clay content (RPIQ = 3.1), followed by sand (RPIQ = 2.7), SOC (RPIQ = 2.5), and silt content (RPIQ = 2.4), resulting from the fusion of the two spectral techniques. The underprediction of very-low and high-clay and SOC-content samples, as well as overpredicted low-content sand samples, was also observed in the combined regression plots (Figure 5).

Specifically, following the combination of LIBS-vis-NIRS spectra, the RMSEP for clay was reduced by 16% and 10% for LIBS and vis-NIRS, respectively, while the RMSEP for silt was reduced by 6% and 17% for LIBS and vis-NIRS, respectively. There was a 6% and 16% reduction in the RMSEP for sand for LIBS and vis-NIRS, respectively. Finally, a 2% and 4% reduction in RMSEP for SOC was recorded for LIBS and vis-NIRS, respectively. From these results, it was evident that data fusion benefited the prediction of texture more as compared to SOC. In another study where LIBS and vis-NIRS were compared, as well as the combined techniques, the authors noted that combining the two techniques did not consistently improve the prediction of total carbon. Instead, LIBS was better at predicting inorganic carbon, while vis-NIRS was better at predicting SOC. The combined technique only improved the prediction of SOC over LIBS and inorganic carbon over vis-NIRS [14]. In a study by Tavares, et al. [35], it was observed that the performance of sensor fusion techniques was dependent on the soil property under investigation. In both of the above-mentioned studies by Bricklemyer, Brown, Turk and Clegg [14] and Tavares, Molin, Nunes, Wei, Krug, de Carvalho and Mouazen [35], 60 soils collected from six fields in Montana, USA, and 102 soils collected from two agricultural fields in Brazil, respectively, were used. Therefore, both datasets covered a smaller scale, in terms of geographical distribution, than in our study. Nonetheless, we still observed varying prediction accuracy depending on the soil property.

#### 3.2.3. Correlation between LIBS and vis-NIRS Predictions

A correlation between LIBS and vis-NIRS predicted values for clay, silt, sand, and SOC was performed to evaluate the degree of the relationship between the prediction models developed for the two techniques.

**Figure 6:**
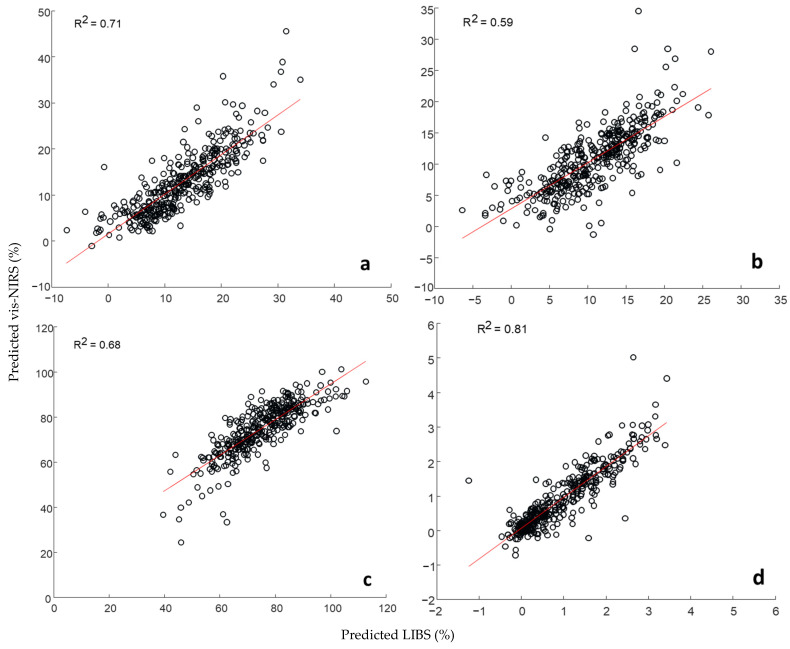
Correlation between LIBS and vis-NIRS predicted values (validation results) for clay (**a**), silt (**b**), sand (**c**), and SOC (**d**).

**Figure 7:**
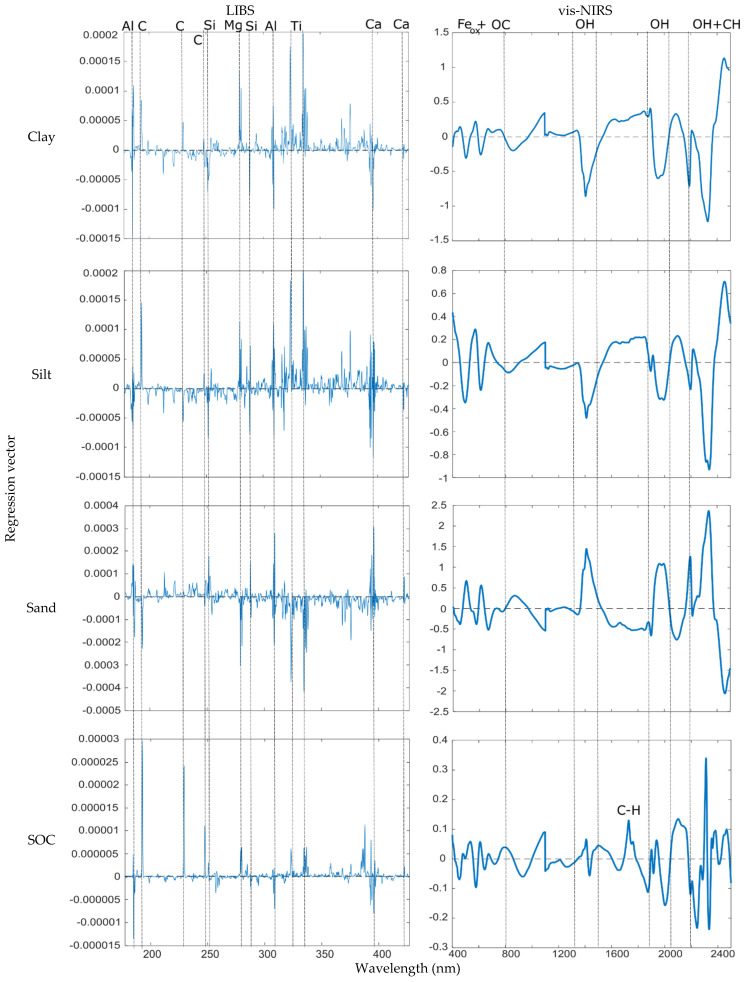
LIBS and vis-NIRS prediction model regression vectors for clay, silt, sand, and SOC, as indicated.

The strongest correlation was observed for the predicted SOC content (R^2^ = 0.81), followed by clay (R^2^ = 0.71), sand (R^2^ = 0.68), and silt (R^2^ = 0.59) (Figure 6). The varying strength of correlation between LIBS and vis-NIRS could be attributed to the performance of the individual single-sensor models, where the more accurate clay, SOC, and sand models exhibited a stronger correlation compared to silt, which had the lowest RPIQ among all the soil properties. The moderate-to-strong correlations also underscore the similarity in terms of the predictive ability of LIBS and vis-NIRS for predicting soil properties [16,36]. Although the techniques are fundamentally different, there could be interrelationships between the variations in soil properties. For instance, clay mineralogy could be a key feature for both LIBS and vis-NIRS prediction models. Additionally, as shown in the regression vector plots (Figure 7), most of the variability of the investigated soil properties is commonly explained by the same features for both techniques, i.e., clay mineralogy, parent rock material, and carbon (or organic matter).

However, as noted by Wangeci, Adén, Nikolajsen, Greve and Knadel [36], the uncertainty of the reference method can influence the prediction accuracy, especially when the reference error is significant. The comparable predictions exhibited by LIBS and vis-NIRS could suggest the high repeatability of the two spectroscopic techniques for predicting soil properties. In other words, the relationship between specific element emission ratios (for LIBS) or absorption bands (vis-NIRS) could provide a more stable measure of the concentration of soil properties in the absence of error-prone reference values. Paradoxically, in addition to soil heterogeneity, the accurate prediction of soil properties could be hampered by the uncertainty of the applied reference method. It remains unclear whether the sample subjected to reference method analysis accurately represents the sample subjected to spectral measurements. This is more of a practical challenge since reference analyses are commonly carried out before spectral measurements. Assuming the sample surface presented for spectral measurements is homogeneous, we still do not guarantee the overall sample homogeneity. This means that, in practice, the sample presented for reference analyses and the sample presented for spectral measurements could be two different samples [37].

#### 3.2.4. Regression Vector Analysis

We referred to the NIST database (for the case of LIBS) on the basis of previous studies (for LIBS and vis-NIRS) to interpret the regression vector plots. For LIBS, the common emission lines identified were related to C, Mg, Si, Al, Ti, and Ca [38]. The Ti 323 and 334 nm emission lines were also identified by Knadel, Gislum, Hermansen, Peng, Moldrup, de Jonge and Greve [16] in a Danish study and could be attributed to the parent rock material for most soils from Denmark (Figure 7).

For vis-NIRS, common absorption bands characteristic to all the investigated soil properties were associated with iron oxides, water molecules, clay minerals, and C-H bonds [11,39,40,41] (Figure 7).

##### LIBS

The prediction of clay was influenced by high positive Ti 323 and 334 nm regression vector scores and positive C 193 nm, C 229 nm, and Mg 279 nm scores. There were also negative Al 185.5 nm and 309 nm, Si 288 nm, and Ca 396 nm regression vector scores. Lastly, there was a less-prominent positive regression vector score associated with C 247 nm.

**Figure 8:**
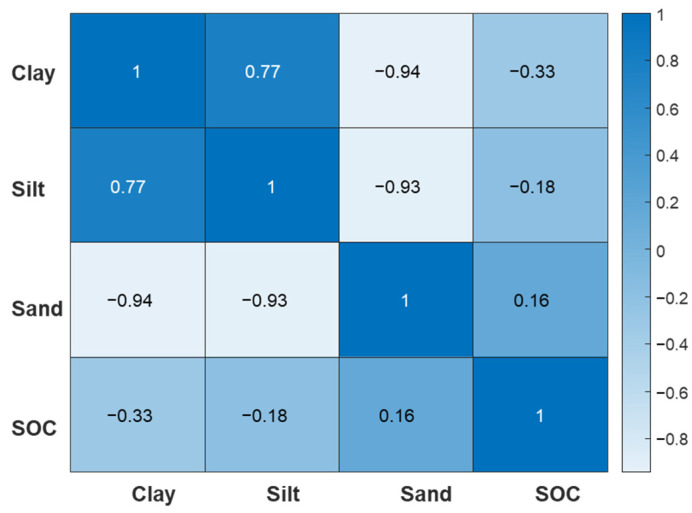
Pearson’s correlation matrix for the investigated soil properties (clay, silt, sand, and SOC). Significant values are presented on a color gradient, ranging from light blue (negative correlations) to dark blue (positive correlations).

Silt variability was influenced by prominent positive C 193 nm, Ti 323 and 334 nm regression vector scores, positive Mg 279 nm, Si 288 nm, Al 309 nm, and Ca 396 nm scores, and negative C 229 nm and Si 251 nm scores. As was the case for clay, silt variability was influenced by a less-prominent positive regression vector score associated with C 247 nm. The influence of similar emission lines on clay and silt variability was also shown by the moderate positive correlation between the two soil properties (Figure 8).

The variability of sand was influenced by high positive Al 309 nm and Ca 396 nm regression vector scores, high negative Ti 323 and 334 nm scores, positive Al 185.5 nm, Si 251 nm and Si 288 nm, and negative C 193 nm and Mg 279 nm regression vector scores. Lastly, there was a less-prominent positive regression vector score associated with C 247 nm. The sand regression vector plot mirrored the clay and silt regression vector plots, as shown by the inverted C 193 nm, Ti 323 and 334 nm, and Ca 396 nm regression vector scores. This was also evident by the strong negative correlation between clay and sand, and clay and silt (Figure 8).

Finally, SOC variability was influenced by prominent positive C 193 nm and C 229 nm regression vector scores, prominent negative Al 185.5 nm, positive C 247 nm, Mg 279 nm, and Ti 323 and 334 nm scores, and negative Al 309 nm, and Ca 396 nm scores. A less-prominent positive C 247 nm regression vector score was also observed in the SOC regression vector plot.

##### Vis-NIRS

The clay variability was influenced by high negative regression vector scores at around 1400 nm and 1900 nm associated with the OH bond, at 2200 nm associated with Al-OH (clay mineralogy), and at 2300 nm related to C-H bonds. Another high positive regression vector score at 2460 nm was visible, which could be related to the C-H band in the functional groups of OM. There were multiple peaks between 470 and 650 nm related to Fe-oxides and OC.

The silt regression vector plot was characterized by a high positive score at 2455 nm and a high negative score at 2300 nm (both associated with the C-H band), negative scores at around 1400 nm and 1900 nm associated with OH, multiple peaks between 470 and 650 nm related to Fe-oxides and OC, and a less-prominent negative score at 2200 nm associated with Al-OH.

The sand regression vector plot reflected the clay and silt plots. The inverse relationship of the properties was also evident in the correlation matrix (Figure 8). There was a prominent positive regression vector score at 2300 nm and another prominent negative score at 2460 nm related to the C-H band. Additionally, there were positive regression vector scores at 1400 nm and 1900 nm associated with OH, at 2200 nm related to Al-OH, and multiple peaks between 470 and 730 related to Fe-oxides and OC.

Finally, the SOC regression vector plot was characterized by high positive and negative regression vector scores between 2300 nm and 2350 nm associated with the C-H band in functional groups of OM and indicative of clay mineralogy. There were positive regression vector scores at 1700 nm associated with C-H, at 2100 nm related to N-H, and at 2200 nm (Al-OH). The multiple peaks occurring between 470 and 750 nm linked to Fe-oxides and OC were also visible. Lastly, there were less-prominent regression vector scores at 1400 nm and 1900 nm associated with OH bonds from water molecules.

#### 3.2.5. Variable Selection

Variable selection using iPLSR was applied to develop independent prediction models for LIBS and vis-NIRS. The results for the investigated soil properties are presented in Table 3. The results obtained after variable selection were also compared based on prediction accuracy with the single-sensor and combined LIBS-vis-NIRS predictions. The RMSECVs and RMSEPs for texture were rounded off to the nearest integer.

The order of performance for the investigated soil properties was largely similar for both techniques, except for vis-NIRS SOC prediction, which performed slightly better than sand. We also noted that for LIBS, iPLSR selected the least number of variables for the prediction of SOC (50 variables), while for vis-NIRS, SOC had the highest number of variables (420 variables) among the investigated soil properties. Variable selection is adopted to exclude wavelength regions that do not describe the variance in the reference data, thereby lowering the interference of noise. While LIBS and vis-NIRS are different techniques, there is a likelihood that the vis-NIRS SOC model had a higher influence of noise as compared to LIBS.

**Figure 9:**
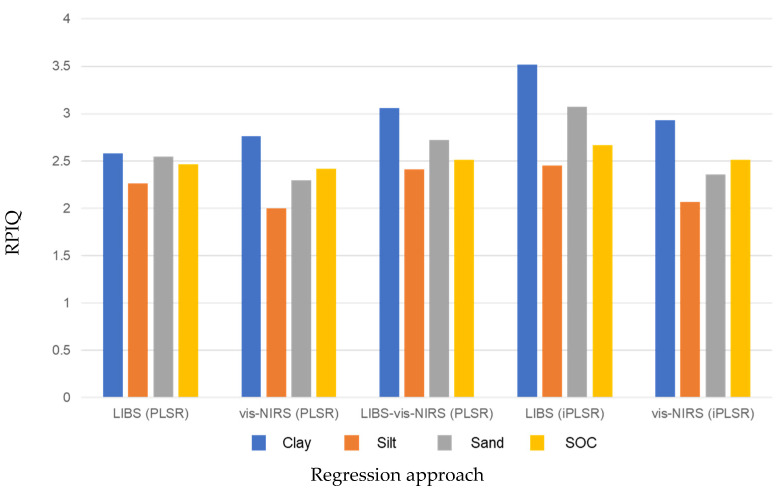
Comparison of performance of the different regression approaches, as assessed using the RPIQ.

Compared to the PLSR (full spectrum) LIBS predictions, there was a 27% and 17% reduction in the RMSEP when variable selection was used for clay and sand, respectively, and a 8% reduction in RMSEP for silt and SOC. For vis-NIRS, there was a 6% and 4% reduction in RMSEP for clay and SOC, respectively, and a 3% reduction in RMSEP for silt and sand. Overall, there was a greater benefit to using variable selection to develop the LIBS prediction models for texture and SOC than vis-NIRS. It was also evident that LIBS performed better (higher RPIQ) than vis-NIRS for all the investigated soil properties (Figure 9).

An additional comparison of the prediction models between variable selection (using iPLSR) and combined LIBS-vis-NIRS showed that independent LIBS models (variable selection) performed better than the combined LIBS-vis-NIRS. In contrast, vis-NIRS (variable selection) models had slightly higher prediction errors compared to the combined LIBS-vis-NIRS models, except for SOC, which had a similar RPIQ (Figure 9).

When applying variable selection algorithms such as iPLSR, there is a risk of selecting wavelength regions that may not be associated with the modeled soil property (noise). For instance, when the number of selected wavelength regions is selected automatically after specifying the interval size, the relevant emission peaks (for LIBS) may be too narrow, or the absorption bands (for vis-NIRS) may be too wide to coincide with the selected interval size, the vice versa is also true. To mitigate this mismatch, various interval sizes are attempted, and the best interval size (lowest RMSECV) is selected. However, this is simply an estimation, which is limited to the number of attempted interval sizes and not necessarily the optimal model.

## 4. Conclusions

In this study, a national-scale dataset was used to compare two spectroscopic methods, LIBS and vis-NIRS, in terms of prediction accuracy. The spectral data from the two techniques were also combined, and prediction models were developed for texture and SOC. For the single-sensor models, LIBS had a higher accuracy for the prediction of silt, sand, and SOC content, while vis-NIRS had a higher accuracy for the prediction of clay content. Nonetheless, when considering the RMSEP and the predicted values from the individual sensors, the performance of both techniques was largely similar. Combining the two techniques improved the prediction accuracy for all the investigated soil properties (except for the LIBS SOC model, which was comparable) as compared to the single-sensor models. Prediction models developed using iPLSR (variable selection) improved the prediction performance of the investigated soil properties for both LIBS and vis-NIRS. There was a notably better performance for the LIBS iPLSR prediction models as compared to both single-sensor and the combined LIBS-vis-NIRS prediction models. This suggests that it could be more beneficial to apply variable selection for LIBS models as compared to coupling with vis-NIRS. Overall, these results demonstrate the capability of both LIBS and vis-NIRS to predict texture and SOC from soils covering different land uses and soil depths with the potential to improve prediction accuracy by combining spectra obtained from both techniques. However, when presented with a choice of the best technique for soil application, other factors, such as the required sample preparation, measurement time, cost, and the accessibility of the instrumentation, should be considered. To enable a fair comparison, future studies should focus on the costs and accuracies associated with different sensor techniques for estimating soil properties. Finally, it is not yet clear why two fundamentally different techniques, LIBS and vis-NIRS, end up with comparable prediction performance. The principal components-based data fusion produced improved prediction results for different soil types of samples containing high sand content applied in this study. Future studies should investigate the effect of the reference method uncertainty on the accuracy of the developed prediction models.

## Figures and Tables

**Table 1:** Descriptive statistics for clay, silt, sand, and SOC in %.

Soil Property	Dataset	Average	Min	Max	SD ^c^	CV ^d^	Q1 ^e^	Q3 ^f^
Clay	Full (n = 1110)	6	1	59	8.38	133	6	17
	Cal ^a^ (n = 739)	6	1	59	8.41	133	6	17
	Val ^b^ (n = 371)	6	2	54	8.31	132	6	17
Silt	Full	5	0	46	6.46	131	6	14
	Cal	5	0	46	6.44	133	6	14
	Val	5	0	40	6.50	129	6	14
Sand	Full	10	12	98	13.63	133	69	85
	Cal	10	12	98	13.61	134	70	86
	Val	10	12	98	13.67	132	68	85
SOC	Full	0.79	0.01	5.92	1.02	128	0.17	1.51
	Cal	0.80	0.01	5.92	1.03	128	0.17	1.51
	Val	0.78	0.02	5.68	1.01	130	0.17	1.45

^a^ Calibration dataset; ^b^ validation dataset; ^c^ standard deviation; ^d^ coefficient of variation; ^e^ first quartile; ^f^ third quartile.

**Table 2:** PLSR cross-validation and prediction results for clay, silt, sand, and SOC.

Property	RMSECV ^a^ %	R^2^ cv ^b^	RMSEP ^c^ (%)	R^2^ pred ^d^	LV ^e^	RPIQ ^f^
LIBS						
Clay	5	0.71	4	0.74	7	2.6
Silt	4	0.67	4	0.67	10	2.3
Sand	7	0.73	7	0.75	10	2.5
SOC	0.53	0.73	0.52	0.73	7	2.5
vis-NIRS						
Clay	4	0.74	4	0.77	10	2.8
Silt	4	0.56	4	0.58	7	2.0
Sand	8	0.67	8	0.69	10	2.3
SOC	0.49	0.77	0.53	0.73	13	2.4
LIBS-vis-NIRS						
Clay	4	0.79	4	0.81	4	3.1
Silt	4	0.70	4	0.71	7	2.4
Sand	7	0.77	6	0.78	7	2.7
SOC	0.47	0.79	0.51	0.75	10	2.5

^a^ root mean square error of cross-validation; ^b^ R^2^ cross-validation; ^c^ root mean square error of prediction; ^d^ R^2^ prediction; ^e^ number of latent variables used for the model; ^f^ ratio of performance to interquartile distance.

**Table 3:** Interval partial least square regression cross-validation and prediction results for clay, silt, sand, and SOC.

Property	NV ^a^	RMSECV ^b^ %	R^2^ cv ^c^	RMSEP ^d^ %	R^2^ pred ^e^	LV ^f^	RPIQ ^g^
LIBS							
Clay	200	4	0.81	3	0.86	14	3.5
Silt	210	3	0.73	3	0.72	13	2.4
Sand	80	6	0.81	6	0.83	13	3.1
SOC	50	0.53	0.74	0.48	0.77	12	2.7
Vis-NIRS							
Clay	330	4	0.77	4	0.8	12	2.9
Silt	360	4	0.63	4	0.61	11	2.1
Sand	130	8	0.69	7	0.71	11	2.4
SOC	420	0.48	0.78	0.51	0.75	11	2.5

^a^ number of variables chosen by iPLS; ^b^ root mean square error of cross-validation; ^c^ R^2^ cross-validation; ^d^ root mean square error of prediction; ^e^ R^2^ prediction; ^f^ number of latent variables used for the model; ^g^ ratio of performance to interquartile distance.

## Data Availability

The raw data supporting the conclusions of this article will be made available by the authors on request.
